# Functional outcomes and survival of patients with oral and oropharyngeal cancer after total glossectomy^☆^

**DOI:** 10.1016/j.bjorl.2019.02.005

**Published:** 2019-03-16

**Authors:** Isabela de Cássia Marins Quinsan, Gustavo Carvalho Costa, Antonio Vitor Martins Priante, Cesar Augusto Cardoso, Caio Lúcio Soubhia Nunes

**Affiliations:** a Universidade de Taubaté (UNITAU), Taubaté, SP, Brazil; b Hospital Regional do Vale do Paraíba (HRVP), Taubaté, SP, Brazil

**Keywords:** Oropharyngeal neoplasms, Glossectomy, Oral neoplasms, Survival analysis, Physiological function recovery, Neoplasias orofaríngeas, Glossectomia, Neoplasias bucais, Análise de sobrevida, Recuperação de função fisiológica

## Abstract

**Introduction:**

Cancer of the oral cavity and oropharynx presents aggressive behavior and its diagnosis is, in most cases, performed in advanced stages. Total glossectomy is a therapeutic option in locally advanced cancer, and the only one in the recurrent or residual disease, after chemoradiotherapy.

**Objective:**

To evaluate the clinical-epidemiological profile, postoperative complications, survival rates and functional aspects of patients with oral cavity and oropharynx cancer after total glossectomy.

**Methods:**

It was a retrospective study where 22 patients were included with oral cavity and oropharyngeal cancer after total glossectomy at the Hospital Regional do Vale do Paraíba, em Taubaté, São Paulo.

**Results:**

All patients were male, with a median age of 57 years, most of tumors are located in the tongue and floor of the mouth and classified as stage IVa. Total glossectomy as initial treatment was performed in 18 and as salvage in four patients. The major pectoralis myocutaneous flap was used for reconstruction in all cases. The main postoperative complication was wound infection and salivary fistula.

**Conclusion:**

Overall survival was 19% and cancer-specific survival was 30.8% in five years. Eight patients were rehabilitated for exclusive oral feeding without the dependence tracheostomy and enteral tube, all with an overall survival greater than 15 months.

## Resumo

**Introdução:**

O câncer da cavidade oral e da orofaringe apresenta comportamento agressivo e seu diagnóstico é, na maioria dos casos, realizado em fases avançadas. A glossectomia total é uma opção terapêutica no câncer localmente avançado e a única no resgate de pacientes com doença residual ou recorrente, após a quimiorradioterapia.

**Objetivo:**

Avaliar o perfil clínico-epidemiológico, as complicações pós-operatórias, as taxas de sobrevida e os aspectos funcionais de pacientes com câncer da cavidade oral e da orofaringe submetidos à glossectomia total no Hospital Regional do Vale do Paraíba, em Taubaté, São Paulo.

**Método:**

Estudo retrospectivo em que foram incluídos 22 pacientes com câncer de cavidade oral e orofaringe submetidos à glossectomia total.

**Resultados:**

Todos os pacientes eram do gênero masculino, com mediana de 57 anos, com tumores principalmente na língua e no assoalho da boca e classificados, em sua maioria, como estádio clínico IVa. A glossectomia total como tratamento inicial foi realizada em 18 e como resgate em quatro pacientes. O retalho miocutâneo peitoral maior foi utilizado para a reconstrução em todos os casos. A principal complicação pós-operatória foi a infecção da ferida operatória e a fístula salivar.

**Conclusão:**

A sobrevida global foi de 19% e a específica por câncer de 30,8% em cinco anos. Oito pacientes foram reabilitados para alimentação oral exclusiva sem a dependência de traqueostomia e ou de dieta enteral, todos com sobrevida global maior do que 15 meses.

## Introduction

Cancer of the oral cavity and oropharynx has an aggressive behavior and the diagnosis is often attained at advanced stages of the disease. In Brazil, 11,200 new cases of oral cavity cancer are expected in men and 3500 in women for the year 2018.^1^

The treatment usually results in esthetic and functional deficits, with a negative impact on quality of life. T4 stage tumors, number of metastatic lymph nodes and male gender are independent predictors of worse prognosis.^2^

Chemoradiotherapy (CRT) has been used for the treatment of advanced tumors aiming at preserving organs, especially in tumors located in the oropharynx. However, total glossectomy (TG) is still a first-line treatment option in extensive tongue cancer, and the only salvage procedure with curative possibilities in patients with residual or recurrent disease, after CRT.^3^

The main surgical challenges of TG are adequate resection of the tumor and reconstruction, aiming to increase the possibilities of cure and to reduce the morbidity related to the functional deficits of the treatment, in order to avoid bronchoaspiration and allow oral feeding without the necessity of using a feeding tube or tracheostomy.^4^ In the literature, the definitive feeding tube dependence in the postoperative period in these patients is as high as 30%.^4^

In the Service of Head and Neck Surgery of Hospital Regional do Vale do Paraíba (HRVP) TG is one of the therapeutic modalities for advanced tumors of the mouth and oropharynx. The evaluation of the functional and survival results of the patients submitted to this type of treatment is important, especially since other therapeutic options with lower morbidity, such as the CRT, are available.

The aim of this study was to evaluate the clinical-epidemiological profile, postoperative complications, survival rates and functional aspects (need for enteral feeding and tracheostomy) of patients with cancer of the oral cavity and oropharynx treated by TG at the HRVP.

## Sample and method

This is a retrospective study carried out through the collection and analysis of data from medical records of 22 patients with oral cavity and oropharyngeal cancer who were treated by TG, whose treatment was initiated from January 2008 to December 2013, in the HRVP in Taubaté, SP, through the Brazilian Unified Health System (SUS). The data collected were recorded in a separate form containing information about the preoperative, treatment, postoperative and clinical follow-up periods. The functional results were evaluated considering the time of decannulation, nasoenteral tube permanence and the need for gastrostomy. The clinical and pathological staging was based on the criteria of the 2009 American Joint Committee on Cancer (AJCC).^5^

Statistical analysis was performed using the software SPSS 13.0 for Windows. Absolute and relative frequency statistics were used to describe the categorical variables and measures of central tendency (mean and median) to describe the numerical variables.

The probability of survival was calculated using the Kaplan–Meier method and the comparison between the curves was performed using the log-rank test. Survival was calculated considering the time in months between the initial treatment and the date of death (in overall survival, death due to any cause and in cancer-specific, from cancer of the mouth and oropharynx), or the date of the last information received. For the calculation of recurrence-free survival, the time in months between the initial treatment and the date of recurrence diagnosis was considered. The statistical significance was set at *p* ≤ 0.05.

The study was approved by the institutional Research Ethics Committee, under approval number 1,681,680.

## Results

Twenty-two patients aged 45–71 years (mean of 57.5 and median of 57 years) were included. All patients were males. At diagnosis, 13 (59.1%) were smoked tobacco and 10 (45.5%) consumed alcohol (Table 1).

**Table 1:** Clinical-epidemiological characteristics of patients submitted to total glossectomy.

Characteristic	*N* (%)
*Age, in years*	
Interval/Mean/Median	45–71/57.5/57

*Gender*	
Male	22 (100)
Female	0

*Risk factors*	
Current smoker	13 (59.1)
Ex-smoker	8 (36.4)
Current alcohol addiction	10 (45.5)
Ex-alcohol addiction	11 (50)

*Tumor location*	
Mouth	7 (31.8)
Oropharynx	2 (9.1)
Mouth and oropharynx	13 (59.1)

*Histological type*	
Squamous cell carcinoma	22 (100)

*Tumor staging*	
T2	4 (18.2)
T3	3 (13.6)
T4a	14 (63.6)
Not informed	1 (4.5)

*Clinical staging*	
CS II	2 (9.1)
CS III	2 (9.1)
CS IVa	15 (68.2)
CS IVb	1 (4.5)
Not informed	2 (9.1)

Most patients, 13 (59.1%), showed involvement of the mouth and oropharynx by the tumor. In 7 (31.8%) cases, the tumor was restricted to the mouth, and in 2 (9.1%) to the oropharynx. All tumors were invasive squamous cell carcinomas (SCC), most of them T4a (14 cases, 63.3%). Regarding the clinical stage (CS), 15 (68.2%) were classified as CS IVa (Table 1).

TG as the initial treatment was performed in 18 (81.8%) patients, and as salvage treatment in 4 (18.2%). Neck dissection was performed in all but 1 case (4.5%), and reconstruction using a major pectoralis myocutaneous flap was used in all cases (Table 2).

**Table 2:** Surgical aspects, follow-up and clinical evolution.

Characteristic	*N* (%)
*Type of treatment*	
Initial	18 (81.8)
Salvage	4 (18.2)

*Neck dissection*	
Unilateral	2 (9.1)
Bilateral	19 (86.4)
Not performed	1 (4.5)

*Type of dissection*	
Radical Classic – RC	1 (4.5)
Radical modified – RM	4 (18.2)
Supraomohyoid	7 (31.8)
RC + supraomohyoid	1 (4.5)
RM + supraomohyoid	5 (22.7)
RC + RM	3 (13.6)
Not performed	1 (4.5)

*Type of reconstruction*	
Pectoralis major	22 (100)

*Hospital length of stay, in days*	
Interval/Mean/Median	6–21/9.9/10

*Postoperative complications*	
Pneumonia	4 (18.2)
Infection/salivary fistula	6 (27.3)
Stroke	2 (9.1)
Lymphatic fistula	2 (9.1)

*Time of follow-up, in months*	
Interval/Mean/Median	1.2–63.6/16.8/9

*Recurrence*	
Yes	12 (54.5)
No	10 (45.5)

*Final status*	
Alive, disease-free	2 (9.1)
Death from cancer	11 (50)
Death from other causes	3 (13.6)
LF, disease-free	3 (13.6)
LF, with recurrence	3 (13.6)

LF, lost to follow-up.

The hospital length of stay varied from 6 to 21 days (mean of 9.9 and median of 10 days). In the postoperative period, 8 patients (36.4%) developed some complications, 4 (18.2%) of them with more than one complication. There were 4 (18.2%) cases of pneumonia, 6 (27.3%) of salivary fistula and or wound infection, 2 (9.1%) of stroke and 2 (9.1%) of lymphatic fistula (Table 2).

Adjuvant radiotherapy was used in 12 (54.5%) of the patients and CRT in 3 (13.6%). One patient (4.5%) with residual disease after salvage TG was submitted to palliative chemotherapy. In this case, complete tumor resection was not possible, and radiotherapy was contraindicated due to previous treatments. He died six months after the surgery, due to the disease.

**Figure 1:**
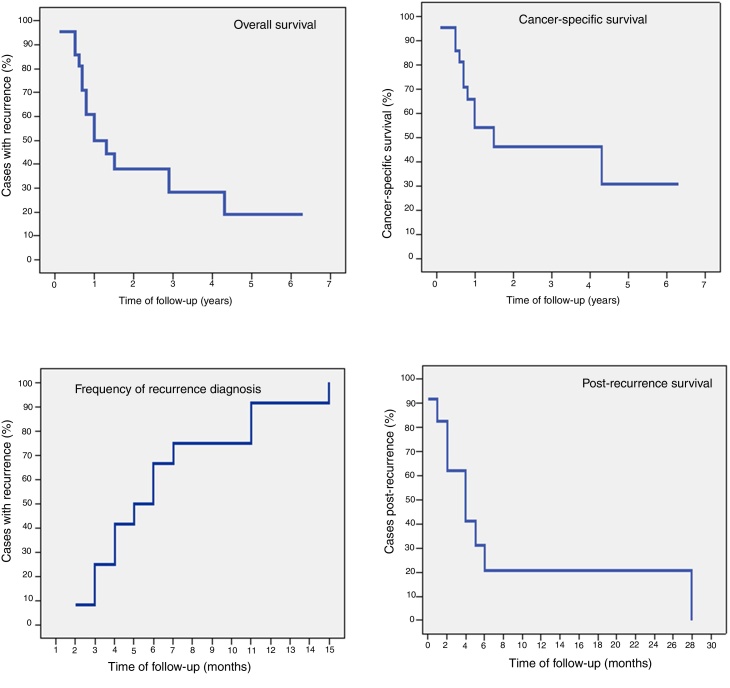
Survival curves.

In the follow-up period, which ranged from 35 days to 63.6 months (mean of 16.8 and median of 9 months), 54.5% of the patients were diagnosed with recurrence. At the time of the last information, only 2 (9.1%) patients were alive and disease-free and 11 (50%) had died from cancer. Six patients were lost to follow-up, of which 3 (13.6%) after recurrence diagnosis and received palliative care (Table 2). Five-year overall survival was 19% (mean of 23.9 and median of 10 months).The 5-year cancer-specific survival rate was 30.8% (mean of 30.3 and median of 15 months). Recurrence-free survival ranged from 62 days to 15.3 months. The longest survival after recurrence diagnosis was 28.5 months (mean of 8.2 and median of 4 months) (Fig. 1).

**Figure 2:**
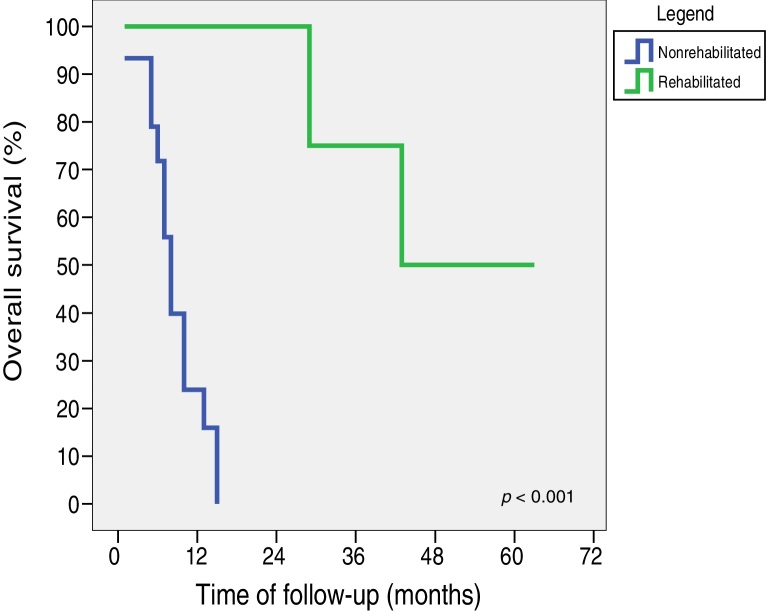
Overall survival curves of rehabilitated and nonrehabilitated patients.

Eight (36.4%) patients did not become dependent on enteral diet neither tracheostomy. Tracheostomy removal was possible in 4 (18.2%) patients, but they maintained the need for partial or total enteral diet. The time to nasoenteral feeding tube removal ranged from 64 to 167 days (mean of 104.7 and median of 92 days), and tracheostomy removal ranged from 17 to 352 days (mean of 92.7 and median of 54.5 days). Conversely, 10 (45.4%) patients required tracheostomy and enteral diet until the date of the last information, with 3 (13.6%) of them being submitted to gastrostomy (Table 3). Patients who were not dependent on the tracheostomy and enteral diet had a significantly longer follow-up and survival than the other patients. Patients who did not require tracheostomy and enteral diet had an overall 5-year survival of 43.8% (mean of 44.3 and median of 43 months). Among those who required tracheostomy or enteral diet, the longest survival was 15 months (mean of 8.7 and median of 8 months) (*p* < 0.001). All patients who survived longer than 15 months were able to progress to oral diet rehabilitation and tracheostomy removal (Fig. 2).

**Table 3:** Functional aspects.

Characteristic	*N* (%)
*Tracheostomy*	
Yes	10 (45.4)
No	12 (54.6)

*Time of removal, in days*	
Interval/Mean/Median	17–352/92.7/54.5

*Enteral diet*	
Yes	15 (68.2)
No	7 (31.8)

*Time of withdrawal, in days*	
Interval/Mean/Median	64–167/104.7/92

*Gastrostomy*	
Yes	3 (13.6)
No	19 (86.4)

## Discussion

TG is one of the treatment possibilities for patients with locally advanced cancer of the oral cavity and oropharynx and remains a controversial procedure,^6^ because although it is technically feasible, it carries a high morbidity.^7^, ^8^, ^9^

Despite advances in the field of oncology for head and neck tumors and the use of CRT for organ preservation, TG in locally advanced cancer of the oral cavity is still the best therapeutic option in most studies.^6^, ^7^, ^8^, ^9^ However, for oropharyngeal tumors, especially those related to human papilloma virus infection, CRT is the standard treatment. On the other hand, in cases of recurrence or residual disease after CRT, surgery is the only treatment modality with possibilities of cure.^10^

From January 2008 to December 2013, 22 total glossectomies were performed in the HRVP. All patients were males. The incidence of these tumors is usually lower in the female gender, due to the fact that women have less exposure to tobacco and alcohol abuse.^11^ In the literature, this proportion is close to 3:1 (male:female),^11^, ^12^, ^13^, ^14^, ^15^ different from that found in our study, probably due to the higher number of patients included in epidemiological studies.

The median age of the patients was 57 years, similar to the findings of other authors.^11^, ^13^, ^14^ Recent studies have included young patients, although the median age at the time of surgery has remained at around 50 years.^4^, ^16^

All tumors were SCC squamous cell carcinoma (SCC). SCC represents approximately 90% of the malignant tumors of the upper aerodigestive tract.^12^, ^13^, ^16^, ^17^, ^18^ Regarding the staging of the primary tumor, 13.6% were T3 and 63.6% T4. 72.7% were CS IV. Advanced CS is considered an independent factor with a worse prognosis^2^; however, those are the cases in which TG is indicated. At the initial clinical evaluation, 4 (18.2%) tumors were classified as T2, two with involvement of the oral tongue and two of the base of the tongue. At this staging, TG is not the treatment; nevertheless, in the intraoperative period, tumor extension was observed to a large part or all the base of the tongue, justifying the use of the procedure for adequate resection of the tumor with free safety margins and disease control. The patient should be aware of the possibility of surgical extension if necessary and the surgeon should be technically prepared to perform the procedure.

Other indications for TG are recurrence or residual disease after surgery or CRT.^10^ In our series, 4 (18.2%) patients were submitted to salvage TG.

TG as the initial therapy in advanced tumors has also been described by other authors.^4^, ^13^, ^14^, ^16^, ^19^ Navach et al. (2012) suggest there are no studies demonstrating the long-term benefits of CRT for the treatment of advanced cancer of the tongue, but only for advanced cases of oropharyngeal and laryngeal tumors.^4^, ^6^ In our study, patients with advanced oropharyngeal cancer also had simultaneous involvement of other sites in the oral cavity, justifying the use of TG as the initial treatment. The two cases in which the tumor was limited to the oropharynx were previously treated with CRT and developed local recurrence, requiring salvage TG.

Stenson et al. (2010) evaluated the difference in overall survival of patients who underwent exclusive CTR or postoperative CTR. In the groups with T3 and T4 tumors, treated with exclusive CRT, overall survival at 5 years was 63%, whereas in the patients treated with surgery followed by CRT, it was 42% in 5 years. These authors did not observe significant differences in survival, suggesting that patients with T4 stage of tongue cancer could be spared from TG as the initial treatment.^20^ However, other factors should be considered and discussed with the patient, including the availability of integrated radiotherapy and chemotherapy services and the patient's clinical conditions to tolerate CRT. Surgical treatment remains the treatment of choice for advanced tumors of the oral cavity.^10^

Of all the patients submitted to TG, the recurrence rate was 54.5%, which is related to the low 5-year overall survival of 19%. According to Barry et al. (2003), who evaluated TG as primary or salvage treatment, local recurrence after RT or surgery for tongue carcinoma is an condition associated with low survival rates. They found a 5-year overall survival rate of 21%, with a recurrence rate of 34.8%.^21^

Eight (36.4%) patients had some type of complication. Surgical wound infection and/or salivary fistula were the most common (27.3%), similar to that found by Vega et al. (2011) which was 23%.^13^ For Navach et al. (2012) the main complication was necrosis of the flap in 10.8% of cases.^4^ In the study by Barry et al. (2003), flap necrosis was observed in 11% of the patients, while salivary fistula was present in 25%.^21^ We did not observe any flap necrosis case in our series.

Regarding the diet evolution, 31.8% of our patients returned to an exclusively oral diet and 13.6% of the patients required a gastrostomy. For Lierop et al. (2008), all patients maintained exclusive oral feeding^3^; however, these authors evaluated only eight patients. On the other hand, in the study by Rihani et al. (2013), only 29% of the patients progressed to an exclusively oral diet.^22^ The non-adherence of some of our patients to the multidisciplinary care, in the ambulatory and home settings, contributed to the non-progression of the diet to oral feeding; however, the main determinant for the non-rehabilitation is the low survival. Studies that included only patients with more than 2 years of follow-up described a return to oral diet in 75% of the cases.^12^, ^13^

Tracheostomy was removed in 54.6% after a median time of 54.5 days, a lower figure than those observed in other studies,^13^, ^17^, ^22^ and similar to what was observed by Sinclair et al. (2011).^23^ For Rihani et al. (2013), 84% of the patients had the tracheostomy removed after 3 years of follow-up.^22^

Dziegielewski et al. (2013) describe 50% of patients with progression to oral diet and 91.6% with progression to tracheostomy removal after one year of follow-up. Patients attending speech therapy sessions had better functional outcomes. The authors also consider that motivated patients with emotional support from their families showed better adherence to medical appointments and rehabilitation sessions with better functional and quality of life results.^17^

The pectoralis major myocutaneous flap was used for the reconstruction of all cases in this series. Some authors have demonstrated better functional results with microsurgical flaps, especially the lateral thigh fasciocutaneous and the rectus abdominis muscle flaps, which allow a greater flap volume to be transferred to the oral cavity and oropharynx, facilitating the reestablishment of the swallowing capacity.^24^, ^25^, ^26^ In our service, although available, microsurgical reconstruction was not utilized in TG cases during the study period.

In our study, we observed that time of follow-up and survival were determinant factors for the best functional results. Rehabilitated patients without the need for tracheostomy and exclusively oral diet had an overall five-year survival of 43.8%, while for those who remained dependent on tracheostomy and/or enteral nutrition, the longest survival was 15 months (*p* < 0.001).

Although TG is an option to be considered in the treatment of advanced oral and oropharyngeal cancer and the only one with potential for cure for post-CRT salvage therapy, the negative impact of treatment on patient quality of life is remarkable, especially regarding the functional aspect.

Initially, most patients remain dependent on either the tracheostomy or enteral diet, with rehabilitation difficulty being influenced by poor adherence to the multidisciplinary treatment, presence of complications, adjuvant therapy, and poor survival. Therefore, it is essential that the patient be motivated and counseled to face a long and challenging rehabilitation period to achieve optimal functional recovery.^25^

## Conclusion

The median age of the patients was 57 years and all patients had exposure to tobacco and 21 to alcohol, either before or at the time of the diagnosis. Tumors were mostly classified as CS IVa. The main postoperative complications were wound infection and salivary fistula. Overall survival was 19.0% and cancer-specific survival was 30.8% at 5 years. Eight patients were rehabilitated for exclusive oral feeding and without tracheostomy dependence, all with survival greater than 15 months.

## Conflicts of interest

The authors declare no conflicts of interest.
